# Death Certification and Murder

**Published:** 1893-10-28

**Authors:** 


// The Hospital

An Institutional Journal of

Science, Medicine, Hygiene, Nursing, and News.

*For Hospitals, Asylums, Infirmaries, Dispensaries, Medical Practitioners, Students, and Nurses.*

Vol. XV.—No. 370.] [Oct. 28, 1893.

### Death Certification and Murder.

The sacredness and safety of human life in any country are not to be measured by the number of murders brought to light and of criminals actually executed, but by the number of persons whose deaths are almost certainly compassed by foul means whether the facts come to light or not. It is estimated that in England there are 7 detected murders for every 10,000 deaths; in Germany, rather more than 6; in France, 8; in Austria, about 9; in Switzerland, nearly 14; in Spain, about 24; in Italy, more than 29. But though in England, taken by herself, there are no more than seven detected murders for every 10,000 deaths, there are 270 deaths in every 10,000 concerning which we do not know how they come about, that is, 270 uncertified deaths in every 10,000.

Now, our whole population in England and Wales is about 26,000,000. Taking the average death-rate for the whole country as 20 per 1,000 per annum, we have a total death-roll for the year of 520,000, and as 2·7 per cent. of these deaths are uncertified, we have a grand total of uncertified deaths for England and Wales alone of 14,040. This, however, by no means shows the actual state of the case for the whole kingdom, for the proportion of uncertified deaths both in Scotland and Ireland is much larger than in England and Wales—in one part of Scotland, Inverness-shire, reaching the astonishing figure of 42 per cent.

These facts and figures, so unexpected, and apparently so incredible, may be accepted as absolutely trustworthy, since they are all taken from authoritative sources; and the more significant of them—that is, those which refer to British murders and uncertified deaths—are abstracted directly from the Report, just published, of the Select Committee of the House of Commons on Death Certification. The two facts which strike one most at the outset are, first, the number of actual murders annually brought to light, namely, 7 in every 10,000 deaths, or the small total of 364; and, secondly, the extraordinary number of uncertified deaths, or possible murders, namely, 14,040. There are in England and Wales alone 14,040 persons buried every year concerning whose death nothing is known. No doctor's certificate has been given in any of these cases; no coroner's inquiry has been held; no official knowledge of any kind has been available. For one reason or another, these 14,040 have been huddled into their graves as quietly, secretly, and expeditiously as possible; but, so far as the medical and judicial authorities of the kingdom know to the contrary, every one of the 14,040 may have been the victim of foul play.

Undertakers, it is said, occasionally see things which are, to say the least, of a very suspicious character. The Select Committee brought out the fact that undertakers, by their own admission, have "sometimes to close the coffin upon what they believe to be a 'foul case,' but which it is not their function to challenge or to examine." Doctors probably see even more than undertakers, and the Committee record that Sir Benjamin Richardson has declared that he does not know "one medical man of extensive practice who, in the course of it, has not met with a case of secret murder." In like manner insurance offices could contribute most important testimony in support of the general contention that many uncertified deaths are uncertified because "murder" is the only true cause which could be assigned. The Report publishes the extraordinary case instanced by the late Dr. Lyons, himself a member of the House of Commons. Dr. Lyons was in attendance upon a patient in Dublin who suffered from bronchitis and asthma. The patient was very ill, and on one occasion had a most alarming paroxysm of difficult breathing during the doctor's visit. The opinion was then expressed that death would some time or other result from such a paroxysm. Next morning a messenger from the patient's house called at Dr. Lyons' house and asked for a death certificate, stating that the patient had died after the doctor's visit. As it happened, the doctor had left his house earlier than usual that morning, and almost at the time when the "friends" of the patient were asking for his death certificate the doctor entered the sick room, and found the reported dead man sitting up in bed, and much better than he had been at the previous day's visit. The object of this manifestly premeditated murder was, it was believed, to obtain the insurance money for which the patient held policies.

It is not necessary, nor have we space, to pile up more evidence on these heads. The facts to be taken into the mind are these, that there are at least 14,040 persons every year who are buried without death certificates, either from doctors or coroners, and that there are positive grounds of the most convincing kind for believing that a great many of the 14,040 are murdered. This is in England and Wales.

What is to be done? First, let us get at all the facts, say those who are new to the investigation. Nothing of the kind! The facts are got at; they have long been known to doctors; they are now brought into full daylight by a Select Committee of the House of Commons which has just closed its sittings; they are published in the Committee's Report; we have here given samples of them. The stage of mere preliminary investigation is passed: the next stage must be the stage of action. What is the state of the law it will be asked? The state of the law is amazing. Ordinary Englishmen have thought that no person could be buried without a doctor's or a coroner's certificate, except secretly and in guilty ways. But what are the facts? "The law," says the Report of the Committee, "does not require either notification or registration as a condition precedent to burial, or to disposal of the body." . . . "Your Committee are much impressed with the serious possibilities implied in a system which permits death and burial to take place without the production of satisfactory medical evidence of the cause of death." . . . "In short, the existing procedure plays into the hands of the criminal classes." These are grave utterances by grave mouths.

What, then, is the remedy? It is perfectly simple, and lies quite ready to hand. It consists, the Select Committee affirms, in competent and universal medical certification. Three orders of officials are required. Those three orders are public registrars, family physicians, and a third order of medical men appointed for this special purpose to act as public "certificators of the cause of death." The members of this third order, though not now certifying causes of death, are doing the work of the public in the capacity of Medical Officers of Health. There is not, therefore, required the creation of a separate order of death certificators. But medical certification to be competent must be the certification of competent medical men who have in every case themselves actually seen the dead body and personally verified the fact of death. This is imperative. Even a family doctor who has attended a given patient through a long illness, must still, as part of a national system, insist upon seeing the body of the reported dead, and personally verifying the fact of death. Only in such a case should the registrar accept the certificate, even of the family doctor. In like manner the public certificator, who, it is proposed, shall be the Medical Officer of Health must, when called upon to certify, compulsorily see the body, and verify the fact, as well as when possible certify the cause, of death. It is well to call to mind that the system proposed by the Select Committee is already in operation in France, and works excellently well. The Public Certificator is an altogether indispensable official on the other side of the Channel. So long as there are in this country 14,040 persons annually who are buried without medical certificates, and many of whom have certainly been murdered, so long are there at least 14,040 convincing reasons why we should at once place competent medical certification in every case of death upon a scientific, compulsory, and absolutely inelusive basis.

